# Functional Metallic Microcomponents via Liquid-Phase Multiphoton Direct Laser Writing: A Review

**DOI:** 10.3390/mi10120827

**Published:** 2019-11-28

**Authors:** Erik Hagen Waller, Stefan Dix, Jonas Gutsche, Artur Widera, Georg von Freymann

**Affiliations:** 1 Physics Department and Research Center OPTIMAS, Technische Universität Kaiserslautern, 67663 Kaiserslautern, Germany; 2 Graduate School Materials Science in Mainz, Erwin-Schroedinger-Str. 46, 67663 Kaiserslautern, Germany; 3 Fraunhofer Institute for Industrial Mathematics, 67663 Kaiserslautern, Germany

**Keywords:** direct laser writing, additive manufacturing, metallic microstructures

## Abstract

We present an overview of functional metallic microstructures fabricated via direct laser writing out of the liquid phase. Metallic microstructures often are key components in diverse applications such as, e.g., microelectromechanical systems (MEMS). Since the metallic component’s functionality mostly depends on other components, a technology that enables on-chip fabrication of these metal structures is highly desirable. Direct laser writing via multiphoton absorption is such a fabrication method. In the past, it has mostly been used to fabricate multidimensional polymeric structures. However, during the last few years different groups have put effort into the development of novel photosensitive materials that enable fabrication of metallic—especially gold and silver—microstructures. The results of these efforts are summarized in this review and show that direct laser fabrication of metallic microstructures has reached the level of applicability.

## 1. Introduction

A large number of applications, e.g., sensors, microelectromechanical systems (MEMS) or high-frequency (HF) antennas, rely on metallic components with on the one hand micrometer-sized features and on the other hand extensions in the millimeter range [1]. These requirements are difficult to meet with traditional fabrication technologies since they are somewhere in the gray zone between subtractive microstructuring or selective laser melting and electron beam lithography or electron/ion beam induced deposition. Not surprisingly, some additive manufacturing technologies have been developed that try to occupy this zone: direct ink writing (DIW, extrusion of metal particle inks, [2]), electrohydrodynamic printing (EHD, electrohydrodynamic ejection of droplets [3]), laser-assisted electrophoretic deposition (LAED, electrophoretic deposition of nanoparticles and laser trapping [4]), laser-induced forward transfer (LIFT, laser-induced ejection of liquid metal droplets [5]), meniscus-confined electroplating (MCE, electroplating from a metal salt solution only at the meniscus formed between pipette and substrate [6]), electroplating of locally dispensed ions in liquid (ELD, electroplating in the interaction region of a dispersed electrolyte and a supporting electrolyte [7]), liquid metal-based direct writing (LMDW, extrusion of a liquid metal from a nozzle while the liquid metal column is stabilized by its oxide skin [8,9]), and direct laser writing (DLW, covered here, for reviews of all these methods see [10,11]). Among these, DLW as a rapid-prototyping fabrication technique offers the greatest versatility, since—a requirement for on-chip structuring capabilities—it does not rely on specific, e.g., conductive, substrates (contrary to EHD, LAED, MCE, and ELD) [12,13]. Furthermore, DLW enables merely unlimited complex structure geometries outperforming most other technologies including LIFT, LMDW, and DIW in this respect.

A disadvantage of DLW is, however, the rather limited range of photosensitive materials available, since up to now most research has been devoted to polymer-based photoresists [14]. In these resists via multiphoton absorption of a tightly focused laser beam, a liquid polymer selectively hardens by cross-linking reactions (negative-tone resists) or a solid polymer selectively turns soluble by, e.g., carboxylic acid generation (positive-tone resists). To the contrary, DLW of metallic structures—first reported in 2006 [15]—is based on photoreduction of dissolved metal ions to neutral metal atoms and subsequent nucleation, growth, and aggregation (introduced in Section 2, for a detailed review of the involved processes see [16]). Since then, a number of groups have fabricated metallic microstructures using DLW either directly [17,18,19,20,21] or indirectly via post-illumination metallization [22,23,24,25,26,27,28,29,30,31,32,33] such as electroless or galvanic growth or plasma sputtering. While indirect processes yield outstanding structure quality, they reduce the versatility of the structuring process since mostly these post-processes are not compatible with on-chip structuring (these processes, e.g., require conductive substrates, do not easily allow for fabrication only on selected parts of the chip or need high temperature treatment) and do not easily allow for fully disconnected structures embedded within a matrix. Therefore, in Section 4 and Section 5 of this review we focus on metallic components with proven functionality directly produced via DLW (introduced in Section 2). The components presented there are all made of noble metals since non-noble metals are not easily produced in a direct laser writing approach due to their unfavourable reduction potential. Accordingly, some post-illumination metallized structures made of non-noble metals are also presented in Section 3. In total, we show that metal direct laser writing (MDLW) of planar structures has reached the level of maturity required for applications while direct fabrication of functional three-dimensional (3D) metallic microcomponents is waiting in the wings.

## 2. Short Introduction to Direct Laser Writing of Metallic Structures

Direct laser writing is an established technology that was first introduced in 1997 [34] and has since been used for the fabrication of highly sophisticated 3D microstructures made of polymer.

**Figure 1:**
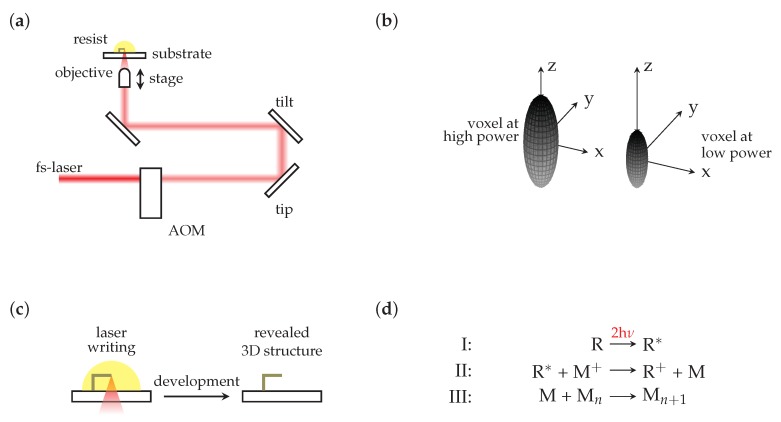
(**a**) Scheme of direct laser writing (DLW) setup: A femtosecond pulsed laser is power modulated by an acousto-optical modulator (AOM). Galvanometer scanning mirrors introduce a tip and tilt and are imaged onto the entrance pupil of a high numerical aperture objective. The objective focuses the beam into a photosensitive material which—in the vicinity of the focal point—selectively hardens (negative-tone resists). The tip and tilt translates the focal point laterally while a stage moves the focal point axially. (**b**) Scheme of the voxel size dependence on incident laser power. Due to the fixed intensity threshold, reducing incident laser power leads to a reduction in voxel size. (**c**) Fabrication process: after direct laser writing, samples are placed in a developer bath for a few minutes to reveal the final structure. (**d**) Principle of metal direct laser writing: in step I, a photoreducing agent (R) is excited by multiphoton absorption. The excited agent donates an electron to metal ions (M${}^{+}$) that are thus reduced (II). The neutral metal atoms nucleate, the seeds grow, and finally aggregate to yield the voxel of a metallic microstructure (III).

A scheme of the setup is shown in Figure 1a. Briefly, near-infrared, femtosecond laser pulses (usually in the range of a few hundreds of femtoseconds) are focused into a photosensitive material by a high numerical aperture (NA) objective lens. Within the photosensitive material via non-linear (e.g., multiphoton) absorption—substantial only in close vicinity of the focal point—a photoreaction is initiated. In combination with a chemical threshold behaviour, this confines the resulting fundamental building block of a structure to a small volume (volume pixel, voxel) that may be changed in size by changing the incident laser power via, e.g., acousto-optical modulators (Figure 1b). By moving the photosensitive material relative to the focal spot, almost arbitrary connected 3D structures may thus be inscribed, with typical writing speeds being of the order of several centimeters per second. A subsequent development or washing step then removes either the unexposed (negative-tone) or exposed (positive-tone) parts and thus reveals the final microstructure (Figure 1c).

To this basic concept a number of technological advances have been added in the past decade [13], e.g., spatial light modulator based DLW [35,36,37,38], high-resolution writing methods such as stimulated emission depletion-inspired DLW [39,40], dip-in DLW [41,42], and multimaterial DLW via an integrated microfluidic system [43]. Furthermore, the diversity of available photosensitive materials has greatly increased, moving away from basic polymer-based photoresists to functional and smart materials such as assessable elastomers [44], various fluorescent polymers [43], and the metal-based solutions addressed here. The mechanism exploited in metal direct laser writing is photoinitiated reduction of dissolved metal ions to metal atoms via electron transfer from a photoreducing agent to the metal ion [16,22,45]. Subsequent nucleation, growth, and aggregation processes in consequence form the fundamental metallic building block of the microstructure (Figure 1d). Mostly, the growth processes are controlled by surfactants and since water is typically used as a solvent, water is often applied in the washing step to remove the unexposed parts.

## 3. Challenges of MDLW and Common Structure Properties

Four major challenges involved with this fabrication process remain: first, the resist is heated by linear absorption of the incident laser by the evolving structure (e.g., particles), which leads to an undesired granular and rough appearance of the structures. Second, contrary to the photopolymerization where absorbed photons initiate a fast chain polymerization, the photoreduction, nucleation, growth, and agglomeration process is rather slow leading to comparably low writing speeds of a few micrometers to a few hundreds of micrometers per second. Third, due to strong interaction of the laser beam with already fabricated features, three-dimensional structures are much more difficult to fabricate by MDLW compared to planar structures. Fourth, due to the unfavourable reduction potential of non-noble metals, MDLW is so far limited to noble metals.

**Figure 2:**
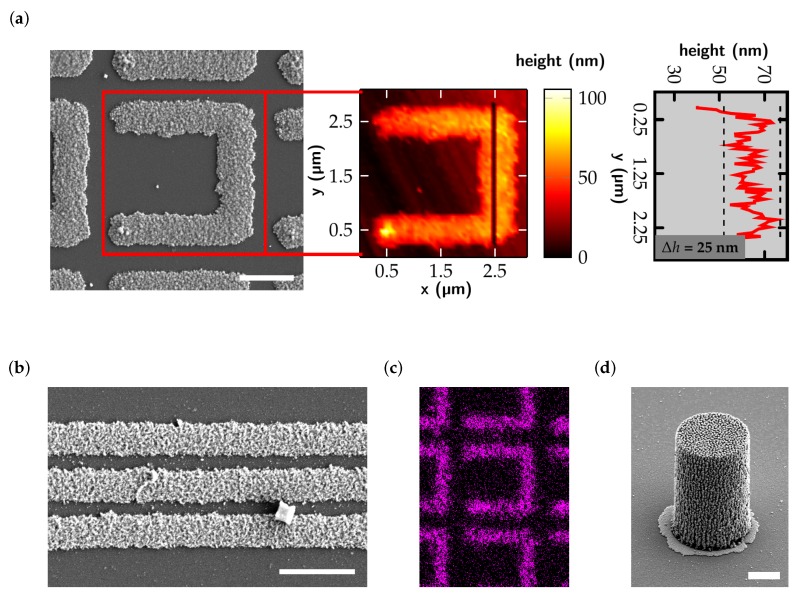
(**a**) Scanning electron micrograph of a planar silver microstructure fabricated by metal direct laser writing (MDLW) (left) and corresponding atomic force microscopy topography measurement (center). The black line indicates the line along which the height plot on the right is taken. The surface roughness of the structure is determined to be around 25 nm peak to valley. (**b**) Scanning electron micrograph of silver lines separated by 600 nm. Scale bars in (**a**) and (**b**) correspond to 1 µm and structures were fabricated with a speed of 1 µm/s. (**c**) Energy-dispersive X-ray spectroscopy (EDX) imaging of silver: the pink color indicates positions at which silver is detected. (**d**) Scanning electron micrograph of a 3D silver monopole with a diameter of 10 µm and a height of 20 µm. The scale bar corresponds to 5 µm.

The low surface roughness of the structures presented in Figure 2a and the applications presented in Section 4 and Section 5 indicate that the first hurdle may be cleared by using surfactants. For example, surface roughness of silver structures is—by now—sufficiently low for optical applications (around a few tens of nanometers peak to valley) and a lateral resolution of 1/600 nm${}^{-1}$ is easily possible (Figure 2b). These values are not far from those that are obtainable for polymeric microstructures fabricated by DLW.

A distinct larger difference is seen for the writing speeds. For example, structures presented in Figure 2 are fabricated with a writing speed of 1 µm/s (one-time scanning), while polymers may be fabricated with several centimeters per second. Clearly, increasing writing speed needs to be focused on in the near future by improving the quantum yield of voxel formation (e.g., by combination of polymerization and reduction). The structures shown in Figure 2 are fabricated using a photosensitive material composed of trisodium citrate as the photoreducing agent and surfactant, silver perchlorate as the silver precursor, and ammonia water as the solvent. The reduction process is initiated by a 780 nm femtosecond pulsed laser focused into the material by an NA = 1.4 oil-immersion objective. The reduction process is very thoroughly as verified by energy-dispersive X-ray spectroscopy (EDX, see Figure 2c) since mostly silver is detected (on the order of approximately 95 weight %).

3D microstructures and 2D structures with resolutions above 2/λ (λ being the wavelength of the structuring beam) are more challenging to produce via MDLW compared to their low resolution 2D counterparts. This is due to the strong interaction of light with metals, e.g., due to destructive interference of incident and reflected wave. Thus, lower wavelengths may help to reduce this effect. In Figure 2d we show a 3D sample monopole structure. This structure is fabricated using the same DLW system as above and a composition of trisodium citrate, silver perchlorate, n-decanoylsarcosine sodium (an additional surfactant), and ammonia water. For fabrication a writing speed of 100 µm/s is used keeping the fabrication time of this solid structure acceptable (around 35 min). The surface of this 3D microstructure is rougher compared to the surface of the planar structures and in the range of half of the wavelength, indicating the strong laser–structure interaction.

**Figure 3:**
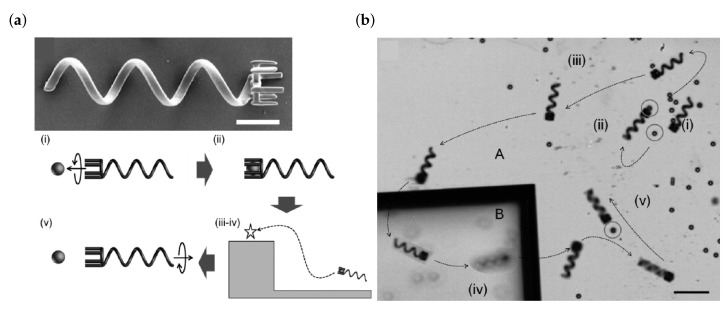
(**a**) Close-up scanning electron micrograph of a helical microswimmer. Scale bar is 10 µm. Below: Schematic of transport capabilities of the swimmer: a rotating magnetic field induces a rotary motion and translation of the swimmer. (**b**) Time-lapse image of the controlled motion as well as cargo pick-up and drop-off of a microswimmer. The scalebar corresponds to 50 µm. Reproduced with permission [28].

Non-noble metal microstructures are not easily produced in a direct laser write approach. The reason for this is the negative reduction potential of these metals, making suitable photoreducing agents a challenging task to find. Some groups have circumvented this issue by indirect methods: a polymer template structure is direct laser written and a subsequent metallization step is performed. A notable example of functional microswimmers for cargo transport is shown in Figure 3a,b [28]. Hereby, the polymer template structure is fabricated using the commercially available photoresists IP-L or SU-8. Next, the swimmers are coated by a Ni/Ti bilayer via electron beam evaporation. A rotating magnetic field (40 Hz, 1.5 mT) enabled precise steering of the swimmers and pick-up as well as drop-off of cargo mit micrometer precision.

## 4. Functional Planar Metallic Microcomponents Fabricated by Direct Laser Writing

Most functional components fabricated by metal direct laser writing are—up to now—planar. Naturally, the majority of these components exploit the electric properties of metals. Therefore, most publications in the field present some kind of wires as proof-of-principle structures [15,17,20]. These type of structures are not the focus of this review but rather components that have a functionality that goes beyond mere electric conduction. We present a few notable exceptions if challenging, unusual substrates, e.g., substrates with topography are used or a complex 3D arrangement of wires is presented.

### 4.1. Electronic Components

**Figure 4:**
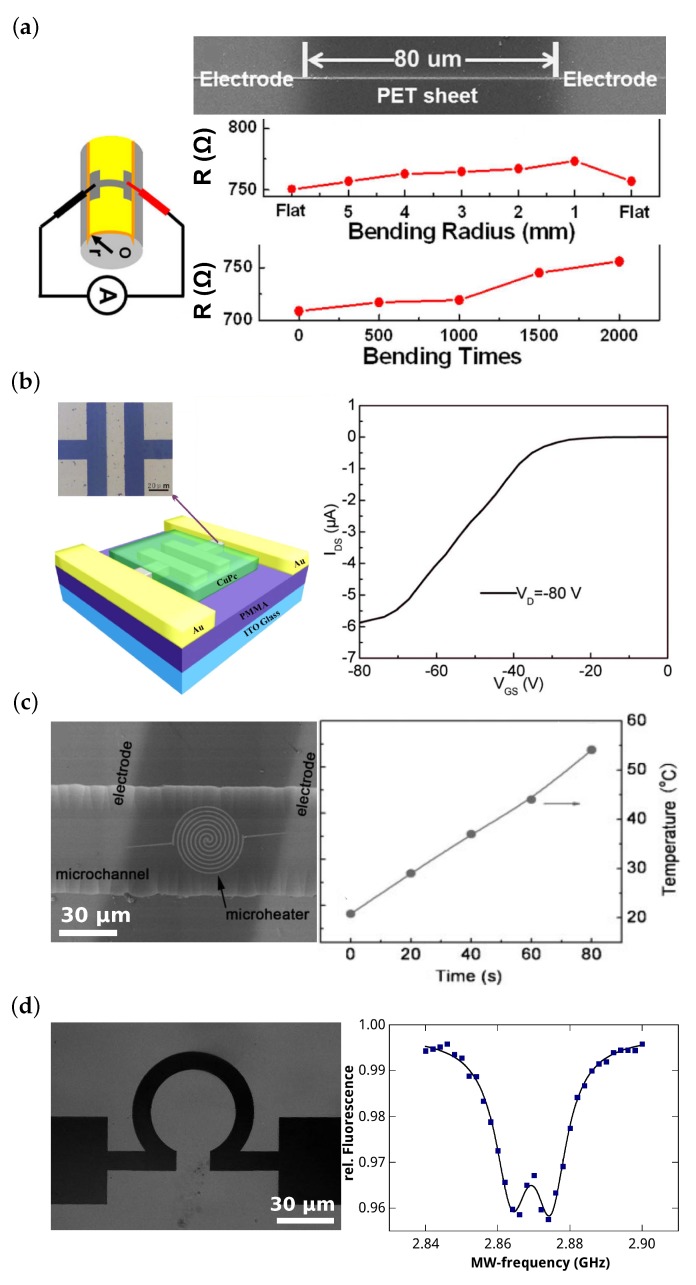
Electric components fabricated by MDLW. (**a**) Scanning electron microscopy (SEM) image of silver wires fabricated on a flexible sheet (top), measurement setup (bottom left), and results (bottom right): resistance versus bending radius and resistance versus bending times. Modified with permission [46]. (**b**) Microscope image of silver source and drain electrodes fabricated by MDLW (top left), scheme of integration in an OFET (bottom left) and measurement of the resulting on-off values (right). Modified with permission [47]. (**c**) SEM image of a silver heating device fabricated inside a microchannel (left) and temperature versus heating time measurement (right). Modified with permission [48]. (**d**) Transmission microscope image of a silver microwave antenna (left) that couples spin-transitions of nitrogen vacancies in nanodiamonds and optical detection of magnetic resonances (right).

One of these exceptions is presented in reference [46] where silver nanowires are fabricated on flexible sheets (Figure 4a). To this end, He et al. employ a two-beam laser writing approach: A Ti:Sa operated at 780 nm wavelength initiates photoreduction while a continuous-wave He-Cd laser (wavelength 442 nm) aids the nucleation process by functioning as an optical tweezer. As focusing lens, a high numerical aperture (NA = 1.42) oil-immersion objective is used. The metal precursor solution is composed of AgNO${}_{3}$ and ammonia water as the silver ion source, n-decanoylsarcosine sodium as the surfactant, and water as the solvent. This solution is sandwiched between a glass and a polyethylene terephthalate (PET) flexible sheet. Despite the hydrophobia of the PET sheet nanowires as small as approximately 150 nm width and a resistivity of $4.6\times 10^{-7}\;\Omega m$ (compared to $1.6\times 10^{-8}\;\Omega m$ bulk silver resistivity) are achieved. The resistance only shows a small dependence on the bending radius for radii larger then 1 mm and the wires are stable enough to survive around 1000 bends without major degradation (see Figure 4a).

MDLW is not restricted to the fabrication of 1D wire structures. In [47], Xu et al. demonstrate fabrication of plane silver electrodes using a silver ion solution doped with 10 nm sized silver seeds. The seeds hereby are added to a composition of AgNO${}_{3}$, ammonia water, and trisodium citrate, the latter being a photoreducing agent and surfactant at the same time. For structuring, a 800 nm femtosecond laser and an NA = 1.45 oil-immersion objective are used. They obtain planar structures with a resistivity of $2.3\times 10^{-7}\;\Omega m$. To demonstrate the feasibility of the method, organic field effect transistors (OFETs) are fabricated (see Figure 4b). To this end, an indium tin oxide covered glass substrate (gate) is coated by a polymethyl methacrylate (PMMA) dielectric layer. On top, two gold electrodes with a gap are fabricated by masked thermal evaporation. Inside the gap the source and drain electrodes are structured by MDLW and an active layer of copper phthalocyanine is subsequently deposited on top by thermal evaporation. As in any field effect transistor, in OFETs the gate-source voltage controls the current flow from source to drain. For a gate voltage of −80 V with the presented device, an on-off ratio of about 200 is achieved.

A similar composition of the photosensitive material as above but not doped with silver seeds is used by Xu et al. for the fabrication of a small heating device inside a microfluidic channel [48] (Figure 4c). The photosensitive solution is drop cast into a channel and the microheater is structured as well as connected to predeposited electrodes by MDLW (using a 790 nm femtosecond laser and an NA = 1.35 oil-immersion objective). The channel is then sealed by a polydimethylsiloxane (PDMS) slab. After supplying a voltage of 1 V to the microheater, the sample could be heated by more than 30 °C within 80 s.

Finally, in Figure 4d we demonstrate the fabrication of a planar silver antenna working around 2.87 GHz. As photosensitive material, we use a composition of trisodium citrate (photoreducing agent and surfactant), silver perchlorate (silver source), and ammonia water (solvent). We employ an NA = 1.4 oil-immersion objective and a 780 nm femtosecond laser for fabrication. Fabricated structures have a specific resistivity less than one order of magnitude larger than bulk silver. This is sufficient for application of such antennas for microwave pumping of nitrogen vacancy centers in nanodiamonds [49,50,51]. Microwave pumping couples the centers’ m = 0 ground state to the degenerate m=±1 levels. External magnetic fields lead to a Zeeman splitting of these levels and fluorescence reduction at two distinct frequencies indicates the population of these spin states, exhibiting smaller fluorescence rates (optically detected magnetic resonance, ODMR). Thus, this antenna-nanodiamond system may be applied as a micrometer-sized optical detector for magnetic fields.

### 4.2. Sensors

**Figure 5:**
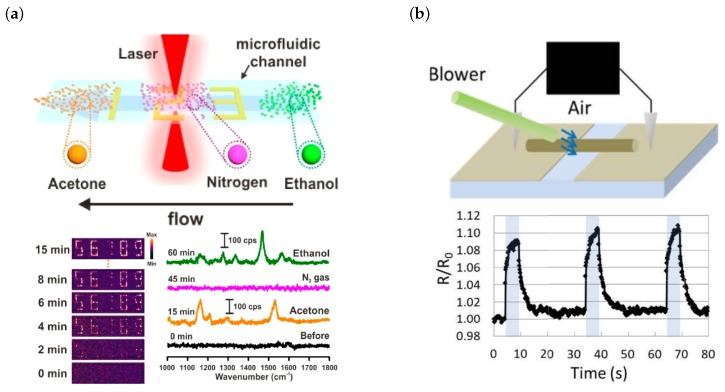
Sensor components fabricated by MDLW. (**a**) PVP-functionalized gold structures fabricated inside a microchannel that enable detection of gaseous 4-MBT, ethanol, acetone, and other gaseous species via surface enhanced Raman scattering: scheme of measurement setup (top), SERS detection of 4-MBT (bottom left), and ethanol as well as acetone (bottom right). Reproduced with permission [52]. (**b**) Scheme of setup (top) to demonstrate the functionality of silver wires for the detection of mechanical forces and measurement of the relative resistance change when applying a small force to the wire (bottom). Reproduced with permission [53].

Two groups have fabricated sensors using MDLW: gas sensors are demonstrated by Lee et al. [52] and mechanical force sensors are demonstrated by Nakajima et al. [53] (Figure 5).

Lee et al. exploit surface-enhanced Raman scattering (SERS) to detect gaseous species [52]. In SERS, the nanoscopic surface roughness of metals leads to a field enhancement via resonant excitation of localized surface plasmons. This excites the Raman modes of the molecule under study. In the discussed publication a SERS enhancement factor of more than $10^{5}$ is achieved with gold-composite microstructures fabricated via MDLW inside a microfluidic channel. The structures are generated from a mixture of HAuCl${}_{4}$ dissolved in ethylene glycol and poly(vinylpyrrolidone) (PVP, playing the role of a surfactant) dissolved in water. Structuring is done using a 780 nm laser and an NA = 1.4 oil-immersion objective. The authors demonstrate detection of gaseous acetone and ethanol which both exhibit low Raman cross sections (Figure 5a) as well as 4-methylbenzenethiol (4-MBT).

Nakajima et al. on the other hand applied MDLW for the fabrication of a mechanical force sensor based on a PDMS-silver-composite structure (Figure 5b) [53]. To this end, they mix photocurable PDMS with silver benzoate in hexane and use a 522 nm wavelength femtosecond laser together with an NA = 0.4 objective for structuring. A 34 µm wide line structure is fabricated between two gold electrodes and its resistivity is determined to be $5.9\times 10^{-1}\;\Omega m$. A mechanical force is then applied to the composite wire via air blowing and the change in electrical resistance is monitored. Due to the bending of the wire and the resulting rearrangement of silver particles inside the wire, the resistance changes with bending radius which in turn depends on the external force. The change in resistance is detectable even though no bending could be observed in a digital microscope and is as large as 10% when air-blowing with 3 kPa.

### 4.3. Metamaterials

In contrast to the components above, most functional components fabricated by MDLW up to now are metallic metamaterials. Metamaterials are artificial materials that lead to material properties that are not found in nature. To this end a subwavelength structure (corresponding to a meta atom) is—usually periodically—repeated. An external field excites internal resonances that depend on the eigenpolarization of the structure. Thus, such media may be described by effective material parameters. The new material properties therefore do not originate from the properties of the base material but rather from the size and geometry of the structure. This enables manipulation of—among others—electromagnetic waves. Metamaterials are applied, for example, as THz-filters.

**Figure 6:**
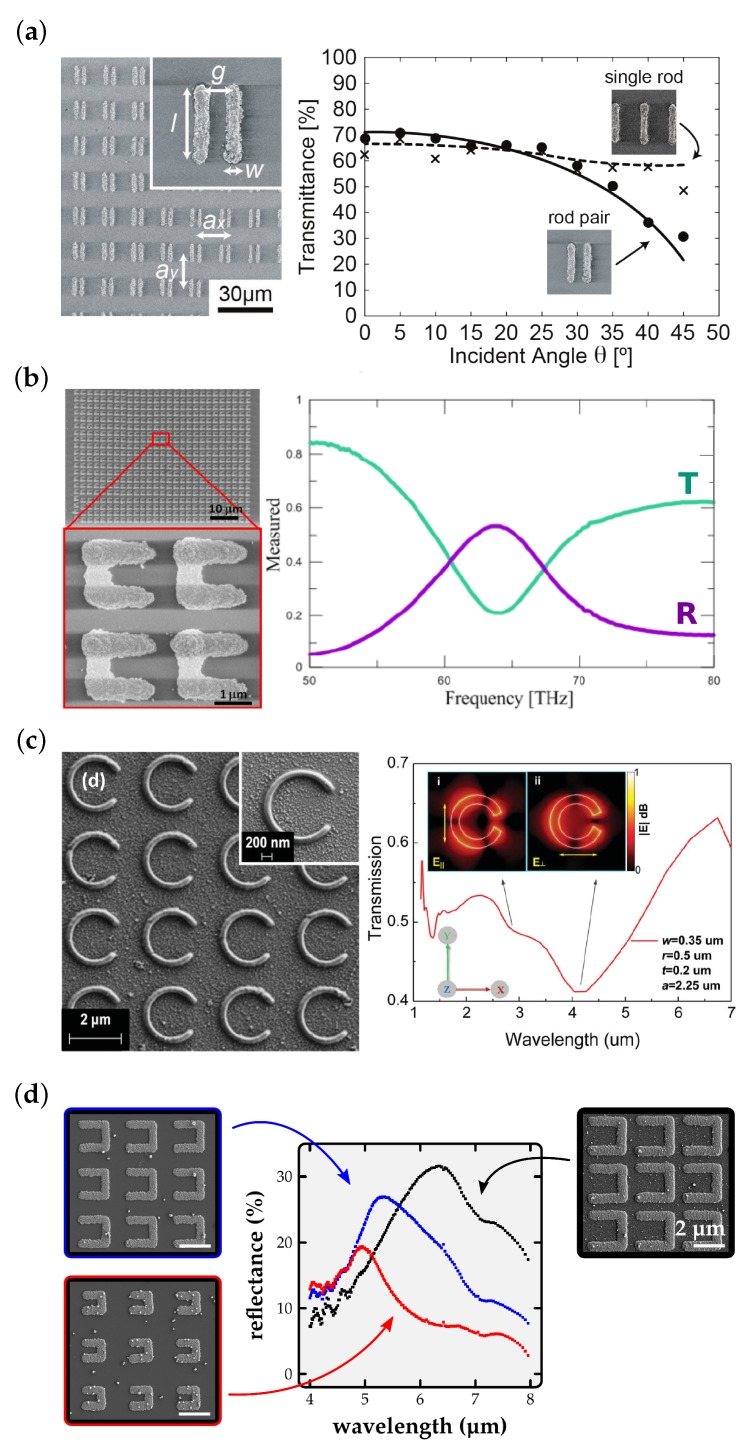
Metamaterials fabricated by MDLW. (**a**) SEM image of a parallel silver rod-based metamaterial (left). Inclined angle transmittance measurement (right): for increasing angle, the magnetic mode of a TE-polarized field at 18 THz increasingly couples to the structure. Reproduced with permission [54]. (**b**) SEM image of gold u-type split-ring-resonators (left) and their transmittance and reflectance spectra (right). A clear resonance is observed at 63 THz. Modified with permission [55]. (**c**) SEM image of a silver c-type split-ring-resonator array (left) and its transmittance spectrum (right). The electric and magnetic resonances are observed. Reproduced with permission [56]. (**d**) U-type silver split-ring-resonator arrays with different leg lengths (left and right) and their corresponding reflectance (center) showing a shift of the resonance towards lower wavelength with lower leg length.

In their 2012 paper [54], Ishikawa et al. show a metamaterial based on a unit cell of two parallel silver rods (length 10 µm, width 1.5 µm, separation 4 µm, see Figure 6a). The unit cell’s dimension hereby is 15 µm × 15 µm. Transmittance measurements over incident angle of the incident transversal electric (TE) polarized light (18 THz) are shown in Figure 6a. The drop in transmittance towards higher angles demonstrates that the magnetic field of the incident light increasingly couples to the magnetic resonance of the metamaterial at higher angles. For fabrication Ishikawa et al. employ a 800 nm femtosecond laser and an NA = 1.42 objective. The material used for MDLW is composed of AgNO${}_{3}$ as the precursor and Coumarin 440 as a photoreducing agent. The produced wires showcase a resistivity of $5.3\times 10^{-8}\;\Omega m$, this is only approximately three times higher than the resistivity of bulk silver.

Lu et al. on the other hand use a gold-based material to fabricate u-type split ring resonator arrays [55]. To this end HAuCl${}_{4}$ is added to a water-based solution of (2-hydroxyethyl)trimethylammonium 5-aminopentanoic (an ionic liquid). As hardware they use a 780 nm femtosecond laser and an NA = 1.45 oil-immersion objective. Resulting structures display a resistivity of $1.7\times 10^{-7}\;\Omega m$ which is of the same order as the resistivity of bulk gold ($2.4\times 10^{-8}\;\Omega m$). In Figure 6b the fabricated split rings and their transmittance spectrum is shown. The spectrum shows the expected electric resonance at 63 THz for the x-polarized wave.

A composition of silver nitrate, ammonia water, dye 2-hydroxy-4’-(2-hydroxyethoxy)-2-methyl- propiophenone, and surfactant n-decanoylsarcosine sodium (NDSS) is used by Tabrizi et al. [56] for the fabrication of very smooth c-shaped split ring resonator arrays. Sample structures and the corresponding transmittance spectrum are shown in Figure 6c. Hereby, the experimentally determined resonance frequency is in decent agreement with theoretical predictions based on the assumption of bulk silver resistivity. This shows that the structure’s conductivity is sufficiently close to bulk conductivity. Contrary to most publications, a visible wavelength is used for fabrication (532 nm, femtosecond pulsed, NA = 1.4 objective).

Gold-composite metamaterial-based polarization rotators are fabricated by Shukla et al. (not shown, [57]). Fabricated structures are composed of polymer and gold. Hereby, the gold source HAuCl${}_{4}\cdot$3H${}_{2}$O, the photosensitive dye AF380, and the polymer SU-8 are dissolved in cyclopentanone. For fabrication a 800 nm Ti:Sa and an NA = 0.85 air objective are used. The authors fabricate a periodic array of Y-shaped noncentrosymmetric structures that—due to the chirality of the unit cell—functions as a polarization rotator.

Finally, in Figure 6d we present our MDLW realization of u-type silver split ring resonator arrays (similar to those published in [16]). As for the antenna structures above, we use a silver perchlorate, ammonia water, and trisodium citrate composition, a 780 nm femtosecond laser and an NA = 1.4 oil-immersion objective for the fabrication of split rings of different sizes. The corresponding non-polarized reflectance measurements are also presented in Figure 6d. The expected blue shift of the reflectance peak for smaller rings and the expected reduced resonance strength for larger relative ring separation is clearly visible.

## 5. Functional 3D Metallic Microcomponents Fabricated by Direct Laser Writing

**Figure 7:**
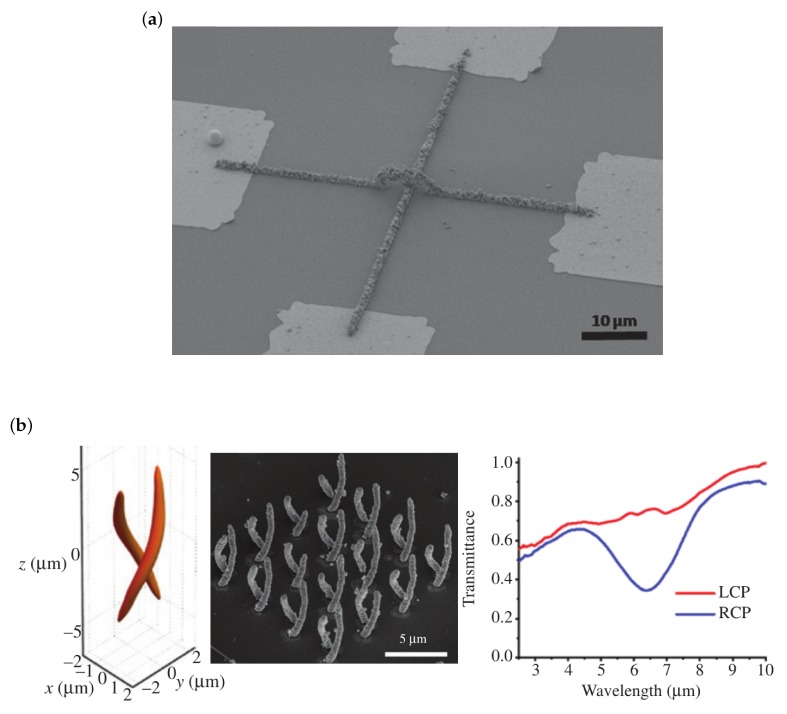
3D components fabricated by MDLW. (**a**) SEM image of two perpendicular gold-composite wires with one of the wires bridging the second one. Reproduced with permission [58]. (**b**) Calculated focal intensity distribution (left) that is obtained by shaping the incident field using a spatial-light-modulator. The shaped focal intensity distribution is used to fabricate the double helix unit cell of a metamaterial in a single shot. An inclined view of the metamaterial is shown in the SEM image in the middle. The chiral metamaterial acts as a polarizer for circularly polarized light with the measured transmittance shown in the right graph. Reproduced with permission [59].

**Figure 8:**
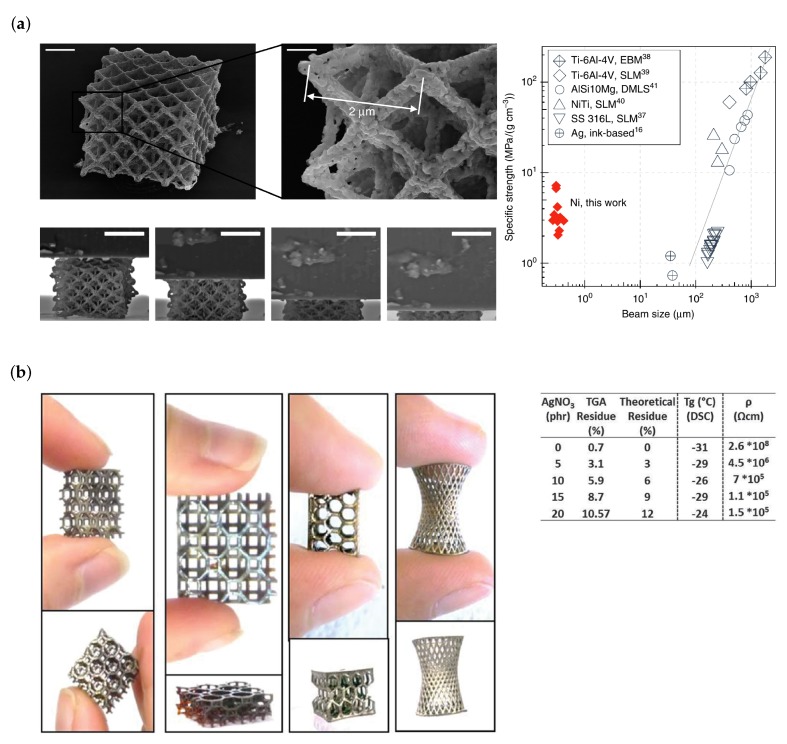
(**a**) SEM image of a nickel microarchitecture after pyrolysis (top left) and close-up (top center). The scale bars correspond to 2 µm and 500 nm, respectively. Bottom left: SEM images during compression test. Scale bars correspond to 5 µm. Right: Diagram of measured specific strength versus structure beam size. Reproduced with permission [60]. (**b**) Photographs of diverse large-scale 3D silver-composite structures fabricated by projection lithography instead of a laser scanning procedure (left images). Right table: Residues measured via thermogravimetric analysis (TGA), T${}_{g}$ values from differential scanning calorimetry (DSC) experiments and resistivity for different photoresist compositions. Reproduced with permission [61].

Very few applications of metallic 3D microstructures fabricated by MDLW are so far found in literature. Among the exceptions are perpendicular but not connected wires [58], metamaterial-based polarizers for circular polarization [59] (see Figure 7a,b, respectively), and high-strength nickel architectures [60] (see Figure 8a). A last example that is—strictly speaking—not fabricated by MDLW but by projection lithography and that shares the same underlying photoreaction is shown in Figure 8b [61].

Similar to Shukla et al. [57], Abargues et al. [62], and Fantino et al. [61], Blasco et al. combine polymer with gold [58]. They obtain conductive 3D wires that are arranged perpendicular to each other without being connected (see Figure 7a). To this end they employ as polymer acrylate-functionalized poly(ethylend glycol), as gold source HAuCl${}_{4}$, as photoinitiator Irgacure 2959, and water as the solvent. A 700 nm femtosecond pulsed laser is used in conjunction with an NA = 1.4 oil-immersion objective for structuring and reduce the repetition rate of the laser from 80 MHz down to 100 kHz (thereby reducing local heating). The resulting wires have a huge resistance in the order of MΩ to TΩ. The resistance reduces dramatically by a thermal annealing step (200 °C, 10 min) to a resistivity of $4.5\times 10^{-7}\;\Omega m$ (bulk gold: $2.4\times 10^{-8}\;\Omega m$) due to rearrangement of atoms of the gold particles and their relocation within the polymer.

The next publication reviewed here is a nice application of spatial-light-modulator-based DLW [59]. The spatial-light-modulator is used to shape the focal field such that it takes the form of a double-helix. This intensity distribution directly, in a single exposure step, generates a double-helix microstructure out of a silver-based material (Figure 7b). This chiral microstructure hereby is the unit cell of a metamaterial that—due to the structure’s circular eigenpolarizations [24]—acts as a polarizer for circular polarized fields (transmitting LCP and suppressing RCP fields). As photosensitive material Liu et al. use the commonly employed composition of trisodium citrate (reducing agent and surfactant), n-decanoylsarcosine sodium (surfactant), AgNO${}_{3}$ (precursor), and ammonia water. Due to the large-sized NDSS molecules the resistivity of fabricated structures is rather large ($3.3\times 10^{-6}\;\Omega m$). For structuring, a 1030 nm femtosecond laser is operated at 100 kHz and the beam is phase modulated by the spatial-light-modulator before being focused into the photosensitive material by an NA = 1.4 oil-immersion objective.

The last two publications reviewed here have fabricated open 3D structures that may, for example, be used as miniature shock absorbers or high volume battery electrodes. To this end, Vyatskikh et al. synthesize a nickel acrylate in a ligand exchange reaction using nickel 2-methoxyethoxide and acrylic acid [60]. To this precursor pentaerythritol triacrylate as an acrylic resin and 7-Diethylamino-2-thenoyl coumarin as a photoinitiator are added to form the metal containing photoresist. Structuring is done at speeds of 4–6 mm/s using a 780 nm femtosecond pulsed laser. In a subsequent pyrolysis step at 1000 ${}^{\circ }$C in argon atmosphere, most organic content is removed (one hour duration). In a further step at 600 ${}^{\circ }$C in forming gas (5% H${}_{2}$, 95% N${}_{2}$) the oxygen content of the structures is reduced (one hour duration). The specific strength of thus produced cellular solids is determined in compression tests and found to be around 2.1–7.2 MPa g${}^{-1}$ for structure beam diameters below one micrometer (Figure 8a). Strictly speaking, this high temperature post illumination step opposes the idea of direct fabrication techniques. However, as mentioned in Section 3, non-noble metals are challenging to produce in a pure direct writing approach.

A slightly different fabrication approach for the fabrication of silver-composite structures is presented by Fantino et al. [61]. They use a commonly available stereolithography and digital light processing (DLP) printer for in-situ polymerization and photoreduction. To this end, they illuminate the photosensitive material with a UV light source (405/365 nm, 1.4 s exposure time per layer). The photosensitive material hereby constitutes silver nitrate (AgNO${}_{3}$), the photocurable oligomer (polyethylene glycol diacrylate, PEGDA), a dye that prevents leaking of light in undesired locations (Reactive Orange 16), the photoinitiator bis-(2,4,6-trimethylbenzoyl) phenylphosphineoxide (Irgacure 819), and the photoreducing agent 2-hydroxy-2-methyl-1-phenyl-propan-1-one (Darocur 1173). 3D microlattice structures with minimum feature sizes of some tens of micrometers are shown in Figure 8b. The resistivity of thus fabricated structures is in the range of 10${}^{5}$ to 10${}^{6}$ Ωcm depending on the silver content of the composite. This high resistivity is typical for composite materials and could probably be reduced by annealing.

## 6. Conclusions

We conclude this review by summarizing the papers addressed here in Table 1.

Clearly, within the last decade a number of functional structures have been fabricated by metal direct laser writing. Most work has been done in the fabrication of planar metamaterials made of silver. However, some groups have fabricated functional gold-based as well as non-planar microstructures. In addition, other applications such as sensors and electrical components are making their appearance. This indicates that MDLW has matured and is becoming a versatile method for on-chip additive fabrication of metallic microstructures. Thus, MDLW paves the road for highly-integrated MEMS, HF-components, and sensors.

## Figures and Tables

**Table 1:** Overview of components fabricated by metal direct laser writing.

Component Type	Material	Substrate	Resistivity	Dim.	Ref.
wires	Ag	flexible PET membrane	$4.6\times 10^{-7}\;\Omega m$	2D	[46]
OFET electrodes	Ag	PMMA	$2.3\times 10^{-7}\;\Omega m$	2D	[47]
microheater	Ag	non-planar microchannel	$1.7\times 10^{-7}\;\Omega m$	2.5D	[48]
GHz antenna	Ag	glass	$1.4\times 10^{-7}\;\Omega m$	2D	own work
					(previously unpublished)
gas sensor	Au-PVP-	microchannel	-	2D	[52]
	composite				
force sensor	Ag-PDMS-	glass, gold electrodes	$5.9\times 10^{-1}\;\Omega m$	2D	[53]
	composite				
metamaterial	Ag	quarz	$5.3\times 10^{-8}\;\Omega m$	2D	[54]
metamaterial	Au	glass	$1.7\times 10^{-7}\;\Omega m$	2D	[55]
metamaterial	Ag	glass	?	2D	[56]
metamaterial	Au-SU8-	glass	-	2D	[57]
	composite				
metamaterial	Ag	glass	$1.4\times 10^{-7}\;\Omega m$	2D	own work
					(previously unpublished)
wires	Au-PEG-	glass	$4.5\times 10^{-7}\;\Omega m$	3D	[58]
	composite		(after annealing)		
metamaterial	Ag	glass	$3.3\times 10^{-6}\;\Omega m$	3D	[59]
nanolattice	Ni	glass	-	3D	[60]
microlattice	Ag-PEGDA-	glass	$1.5\times 10^{-3}\;\Omega m$	3D	[61]
	composite				

## Abbreviations

The following abbreviations are used in this manuscript:

MEMS	Microelectromechanical systems
HF	High-frequency
DIW	Direct ink writing
EHD	Electrohydrodynamic printing
LAED	Laser-assisted electrophoretic deposition
LIFT	Laser-induced forward transfer
MCE	Meniscus-confined electroplating
ELD	Electroplating of locally dispensed ions in liquid
DLW	Direct laser writing
MDLW	Metal direct laser writing
3D	Three-dimensional
NA	Numerical aperture
AOM	Acousto-optical modulator
EDX	Energy-dispersive X-ray spectroscopy
PET	Polyethylene terephthalate
OFET	Organic field effect transistor
PMMA	Polymethyl methacrylate
SEM	Scanning electron microscopy
PDMS	Polydimethylsiloxane
ODMR	Optically detected magnetic resonance
SERS	Surface-enhanced Raman scattering
4-MBT	4-methylbenzenethiol
PVP	Poly(vinylpyrrolidone)
TE	Transversal electric
NDSS	N-decanoylsarcosine sodium
2D	Two-dimensional
TGA	Thermogravimetric analysis
DSC	Differential scanning calorimetry
DLP	Digital light processing
PEGDA	Polyethylene glycol diacrylate
